# The Importance of the Neutrophil-Lymphocyte Ratio in Patients with Idiopathic Peripheral Facial Palsy

**DOI:** 10.1155/2015/981950

**Published:** 2015-12-02

**Authors:** M. Mustafa Kiliçkaya, Mustafa Tuz, Murat Yariktaş, Hasan Yasan, Giray Aynalı, Özkan Bagci

**Affiliations:** ^1^Department of Otolaryngology, Medical Faculty of Suleyman Demirel University, 32100 Isparta, Turkey; ^2^Department of Medical Genetics, Medical Faculty of Suleyman Demirel University, 32100 Isparta, Turkey

## Abstract

*Objective*. The purpose of this study was to investigate whether or not there was a correlation between the neutrophil-to-lymphocyte ratio (NLR) value and the severity of idiopathic peripheral facial palsy (IPFP) and to determine whether or not NLR could be used as an early predictive parameter in the prognosis of IPFP patients.* Material and Method*. This retrospective study was conducted on 146 patients who were diagnosed with IPFP. The control group comprised 140 patients. Patients with IPFP were categorized according to the House-Brackmann grading system (HBS). The NLR value was obtained by dividing the neutrophil value by the lymphocyte value.* Results*. In the IPFP group, the mean NLR value was 3.63 ± 2.74 and, in the control group, 1.84 ± 0.78. The mean NLR value was significantly higher in IPFP patients than in the control subjects (*p* < 0.0001). The mean NLR value in group A (Grades I-II ) was 2.61 ± 2.28, in group B (Grades III-IV) 3.22 ± 2.65, and in group C (Grades V-VI) 10.69 ± 6.30.* Conclusion*. We determined that as the severity of IPFP increased, the NLR value increased. The NLR value can be used as a prognostic factor in the early prediction of IPFP prognosis.

## 1. Introduction

Idiopathic peripheral facial palsy (IPFP) is a disease seen in 20–30 people per 100,000 per year [1]. IPFP, which generally affects a single side of the face, is an acute IPFP [2]. Although the pathology is not fully known, viral infections, vascular ischaemia, and inflammation are widely held responsible [2]. Of viruses, varicella-zoster virus (VZV) and herpes simplex virus (HSV) are most commonly blamed [3, 4]. There are several systems to evaluate the severity of IPFP, of which the most widely used is the House-Brackmann grading system (HBS). Grading is made from I to VI with Grade I as normal and Grade VI as total palsy. On the HBS scale, Grades I-II are stated as mild paralysis, Grades III-IV as moderate paralysis, and Grades V-VI as severe paralysis with a poor prognosis [5].

Some studies, if not all, recognize diabetes, hypertension, hypercholesterolemia, old age, and paralysis severity as factors associated with poor prognosis of IPFP [6, 7]. In the early stages of this disease, many tests are applied to predict prognosis, including stapes reflex (SR), electromyography (EMG), nerve excitability test (NET), and electroneuronography (ENoG) and blink reflex tests [8]. White blood cells (WBC) and the number of subtypes such as neutrophils in particular are accepted as classic inflammatory markers. Several studies have reported that the NLR could be used as a marker of inflammation. In addition, it has been used as an indicator of prognosis in some diseases such as cardiac diseases and neoplasia [9, 10].

Our aim was to investigate whether or not there was a correlation between the severity of IPFP, which is thought to be related to inflammation and a high level of the NLR value and to determine whether or not NLR could be used as an early predictive parameter of prognosis in patients with IPFP.

## 2. Materials and Methods

This retrospective study was performed on 146 patients (76 males, 70 females) who were diagnosed with IPFP in the Otorhinolaryngology Clinic of Suleyman Demirel University Faculty of Medicine Hospital between February 2009 and January 2015. Ethics board approval for this study was granted by the Suleyman Demirel University Medical Faculty, in accordance with the Declaration of Helsinki. Patients were excluded with diseases which could affect the NLR, such as active ear disease, acute inflammation or infection, acute or chronic renal failure, chronic liver disease, heart disease, chronic obstructive pulmonary disease, neurological disorders, or neoplasm. We included patients with diabetes, hypertension, and hypercholesterolemia in the study. The control group comprised 140 patients (70 males, 70 females) with no active ear disease or facial nerve pathology who were undergoing preoperative tests in the ENT polyclinic for septoplasty or myringoplasty operations. The study group and control group were retrospectively scanned for the neutrophil and lymphocyte values. More than half of the patients had their facial paralysis severity grades (on early referral and control examination) recorded in patient files based on the House-Brackmann grading system. Some patients' grades were not indicated. We provided detailed descriptions of their mimic muscle movements, and found their HBS grade based on such data. All patients included in the study referred to our clinic in the first three days of paralysis, and we measured, as a routine, their complete blood count on early referral. In the case of facial paralysis, instead of the grade measured on early referral, we took the grade that corresponded to the most advanced stage of paralysis as initial grade, for paralysis may grow worse in a couple of days. In the patient follow-up, those with facial motor deficit at the end of 1 year were accepted as patients with permanent motor deficit. Follow-up period for the patients included in the study varied between six months and six years. The group composed of twelve patients with permanent facial motor deficit had a minimum follow-up period of two years. We reinvited those patients with permanent facial motor deficit to a control examination in order to determine their paralysis grades based on the HBS. The IPFP patients were classified according to the HBS as follows: a diagnosis of Grades I-II paresis was made in 61 patients (41.78%), Grades III-IV in 72 (49.31%), and Grades V-VI in 13 (8.90%). Grades I-II patients were designated as group A, Grades III-IV patients as group B, and Grades V-VI as group C. The NLR values of groups A, B, and C were calculated and statistical analysis was applied. All the patients were treated with steroids or steroids+antiviral medication. After 15 days, additional physical therapy was applied to some patients in whom paralysis continued.

### 2.1. Hematological Analysis

Blood samples were collected into tubes containing calcium EDTA and an automated blood cell counter was used for complete blood count (CBC) test measurements (Beckman Coulter LH 780 Hematology Analyzer, USA). The NLR value was obtained mathematically by dividing the neutrophil value by the lymphocyte value.

### 2.2. Statistical Analysis

In the statistical analysis, the *t*-test was used to evaluate the mean NLR in the IPFP and control groups, Kruskal-Wallis analysis was applied to the differences between groups A, B, and C to determine from which group the difference originated, and Bonferroni correction was performed. Mann-Whitney *U* test was used to compare differences between the IPFP patients with complete recovery and those with permanent motor function deficit. A value of *p* smaller than 0.05 was considered significant.

### 2.3. Results

**Figure 1:**
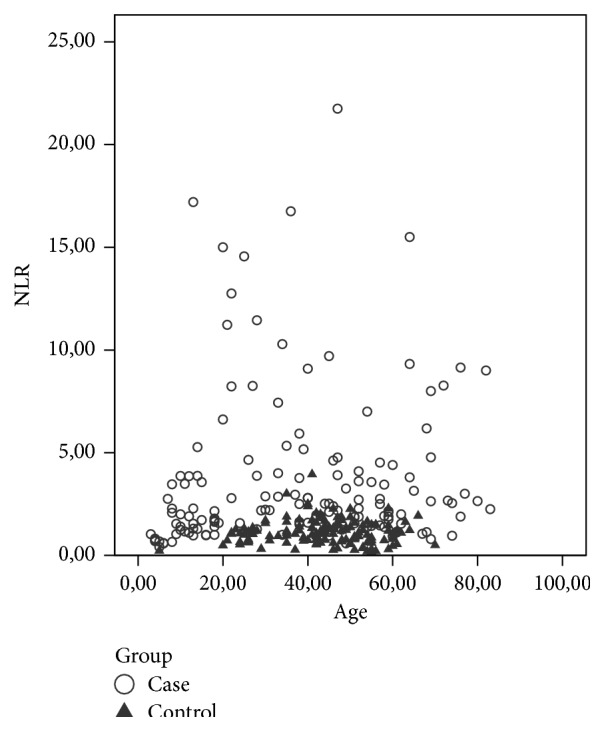
The distribution according to age of NLR in case and control groups.

The average age of the patients with IPFP was 41.62 ± 22.32 and that of the control group subjects was 43.64 ± 11.68 years. Tables 1 and 2 present the age distribution and average age figures for groups A, B, and C. It was shown that NLR was not affected by age in control groups (Figure 1). One-way ANOVA analysis revealed no statistically significant difference between the groups in terms of average age (*p* = 0.560). All patients had unilateral palsies: 72 cases were right-sided and 73 left-sided. The mean NLR value was 1.84 ± 0.78 in the control group and 3.63 ± 2.74 in the IPFP group. The average NLR value was found to be remarkably higher in patients with IPFP than in the control group (*p* = 0.000). No statistically remarkable difference was determined between IPFP patients with DM (26 patients, 17.80%) and IPFP patients without DM (122 patients, 83.56%) (*p* = 0.547). There was no statistically significant difference between the groups in terms of patients with DM (*p* > 0.05) (Table 3).

**Figure 2:**
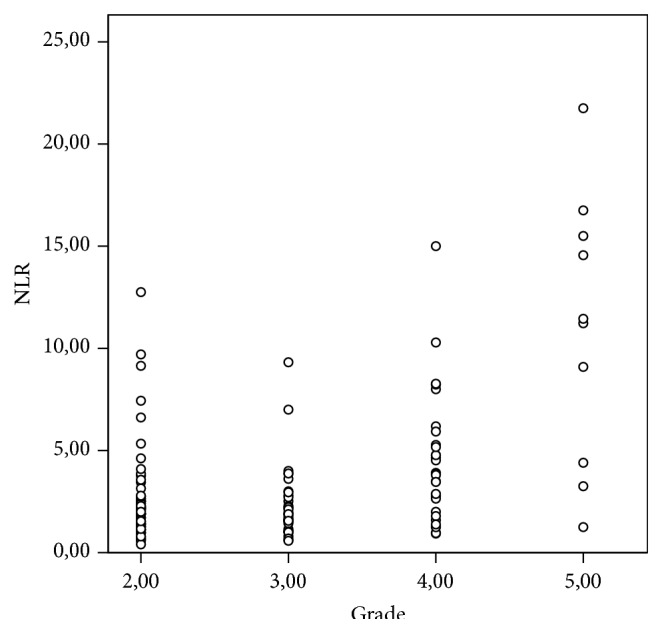
The distribution according to grade of NLR in IPFP.

The mean NLR value in group A was determined as 2.61 ± 2.28, in group B as 3.22 ± 2.65, and in group C as 10.69 ± 6.30. According to the results of the Kruskal-Wallis analysis, the difference between the mean NLR values of the 3 groups was statistically highly significant (*p* < 0.0001). As the severity of the paralysis increased in the IPFP patients in this study, the NLR value increased (Figure 2). To determine from which group the difference originated, Bonferroni correction was applied and it was determined that the difference originated in group C (*p* = 0.000, *p* = 0.000, and *p* = 0.136, groups C-A, groups C-B, and groups A-B, resp.).

At the end of the follow-up period, 12 of 146 patients were determined with permanent facial motor deficit. The mean NLR value of 8 (66.66%) of these 12 patients was found to be high compared to the control group (Table 4). The mean NLR of the IPFP patients with complete recovery was 3.44 ± 2.50 and in the 12 patients with permanent facial motor function deficit the mean NLR value was found to be 5.55 ± 4.41. According to the Mann-Whitney *U* analysis, there was no as statistical remarkable difference (*p* = 0.11).

## 3. Discussion

Rapid response to acute inflammation is provided by leukocytes and plasma proteins. The basic leukocytes in acute infection are neutrophils. Acute inflammation is triggered by events such as bacterial or viral infections, trauma, tissue necrosis, and immune reactions. In the response to viral infections of the body, the T-lymphocytes play a greater role [11]. In the current study, the mean NLR values of the patients with IPFP were significantly higher than those of the control group and it was also determined that as the severity of IPFP increased, the NLR value increased. In some studies, it has been reported that when there is an increase in the initial degree of IPFP the prognosis worsens [12]. Therefore, it can be considered that patients with a high NLR value could have a poor prognosis. In addition, generally the disease progresses within a few days from the initial symptoms to maximal paralysis [5]. Certain electrophysiological tests, such as EMG and ENoG, are employed to estimate IPFP prognosis. However, they are not very reliable in the first week of paralysis [13, 14]. The NLR value may be high in blood tests made on the first day. Therefore, the NLR value on the first day of presentation may be a significant indicator in the prediction of prognosis.

Some studies report increased NLR associated with age and smoking [15]. In our study, we found that NLR was not affected by age. As smoking status was not recorded for all patients in patient files, we could not examine possible impacts of smoking on NLR.

There are studies in literature which have stated that steroid use is the most effective treatment in the early stage for IPFP patients [12, 16]. Just as it has been advocated that there is no benefit from antiviral medications in IPFP treatment, some clinical studies have reported that the combination of steroids with antiviral drugs was more beneficial [17, 18]. Considering that the prognosis could be poor for patients with a high NLR value in the blood tests made on the first day, it may be useful to inform the patient and immediately start steroid+antiviral therapy and the importance of this therapy for the patient can also be suggested.

## 4. Conclusion

NLR is accepted as a new potential marker of inflammation. As the severity of the paralysis increased in the IPFP patients in this study, the NLR value increased and the majority of the patients with permanent facial motor function deficit were determined to have a high NLR value. Therefore, the NLR value can be used as an early predictive prognostic factor of IPFP.

## Figures and Tables

**Table 1:** The age distribution of groups A, B, and C.

Age	Group A	Group B	Group C
≤15	15 (24.59%)	18 (25.35%)	1 (7.69%)
16–45	16 (26.22%)	27 (38.02%)	5 (38.46%)
46≤	30 (49.18%)	26 (36.61%)	7 (53.84%)

**Table 2:** The average age of groups A, B, and C.

	Mean age ± Std. deviation
Group A	38.90 ± 22.77
Group B	36.04 ± 21.77
Group C	42.30 ± 17.43

**Table 3:** The distribution of patients with diabetes mellitus.

	The patients with diabetes mellitus
Group A (*n* = 61)	15 (24.59%)
Group B (*n* = 72)	10 (13.88%)
Group C (*n* = 13)	2 (15.38%)

**Table 4:** The initial NLR values in the 12 patients with permanent facial motor function deficit.

Patients	Age	Initial grading of HBS	Final grading of HBS	Initial NLR value
Case 1	51	Grade II	Grade II	1,87
Case 2	67	Grade III	Grade II	1,05
Case 3	57	Grade IV	Grade II	**4,51**
Case 4	32	Grade IV	Grade II	**3,25**
Case 5	20	Grade IV	Grade II	**15,00**
Case 6	13	Grade IV	Grade II	0,96
Case 7	79	Grade IV	Grade II	**2,64**
Case 8	11	Grade IV	Grade II	**3,48**
Case 9	18	Grade IV	Grade II	1,78
Case 10	21	Grade V	Grade II	**11,22**
Case 11	13	Grade VI	Grade II	**17,00**
Case 12	47	Grade VI	Grade IV	**3,56**

Mean				**5.55 ± 4.41**

NLR: Neutrophil-to-lymphocyte ratio. HBS: House-Brackmann grading system.
